# Could the Majority of the Greek and Cypriot Population Be Vitamin D Deficient?

**DOI:** 10.3390/nu14183778

**Published:** 2022-09-13

**Authors:** Souzana E. Xyda, Kalliopi Kotsa, Argyrios Doumas, Emmanouil Papanastasiou, Alexandros A. Garyfallos, George Samoutis

**Affiliations:** 1 Medical School Department, Faculty of Medical Sciences, University College London, Gower Street, London WC1E 6BT, UK; 2 Division of Endocrinology and Metabolism, 1st Department of Internal Medicine, AHEPA Hospital, Medical School, Aristotle University of Thessaloniki, 54124 Thessaloniki, Greece; 3 Department of Radiology and Medical Informatics, AHEPA Hospital, Medical School, Aristotle University of Thessaloniki, 54124 Thessaloniki, Greece; 4 Laboratory of Medical Physics and Digital Innovation, Medical School, Aristotle University of Thessaloniki, 54124 Thessaloniki, Greece; 5 4th Department of Internal Medicine, Hippokration University Hospital, Medical School, Aristotle University of Thessaloniki, 54124 Thessaloniki, Greece; 6 Department of Primary Care and Population Health, University of Nicosia Medical School, Nicosia 2408, Cyprus

**Keywords:** vitamin D, cholecalciferol, Cyprus, Greece, vitamin D deficiency, vitamin D insufficiency, vitamin D status

## Abstract

OBJECTIVE: Hypovitaminosis D is prevalent in epidemic proportions in many developed countries. The aim of this study is to investigate the prevalence of adequate 25-hydroxyvitamin D [25(OH)D] levels in two Mediterranean countries, Greece and Cyprus. METHODS: Data such as 25(OH)D, the month of blood sample collection, and demographic information were blindly collected from 8780 Greek and 2594 Cypriot individuals over 5 years. Comorbidities were also recorded for 839 Greek subjects. Univariate and multivariate analyses were used to examine the relationship between these variables and 25(OH)D levels. RESULTS: In the samples studied, 72.7% of the Greek and 69.3% of the Cypriot population sample had inadequate levels of 25(OH)D. The mean level for the Greek subjects was 25.1 ng/mL and for Cypriots 25.8 ng/mL. For both samples, only month and gender were significantly associated with 25(OH)D levels, and the highest mean levels were recorded in September. For the recorded diseases, the lowest levels were recorded in sickle cell anaemia 13.6 ± 10.2 ng/mL, autoimmune diseases 13.0 ± 8.4 ng/mL, and cancer 22.6 ± 9.5 ng/mL. CONCLUSIONS: The prevalence of vitamin D deficiency is paradoxically high in both Mediterranean countries.

## 1. Introduction

Vitamin D plays a pivotal role in bone health and calcium metabolism [1], but it also has a plethora of biological actions through its function as a steroid hormone; it has been suggested to reduce the risk of cancer, autoimmune diseases, and infectious diseases [2]. The majority of vitamin D is synthesised through a UV-mediated reaction in the skin [3,4] and 25-hydroxyvitamin D [25(OH)D] is its major circulating form [5]. For the purposes of this study, the terms vitamin D and 25(OH)D will be used interchangeably.

Vitamin D status and vitamin D3 supplementation affect broad gene expression in humans [6,7]. Vitamin D3 supplementation alters by a minimum of 1.5-fold the expression of 291 genes of more than 160 pathways linked to cancer, cardiovascular, and autoimmune disease [7]. A revolution in this field was the discovery of the vitamin D receptor (VDR)—the receptor through which vitamin D exerts its modulatory effects in the cells—in non-renal compartments, including cells of the adaptive and innate immune system [1,2,8]. VDR can regulate the transcription of antimicrobial peptides [9], protecting the respiratory [1] and gastrointestinal tracts [10] against a variety of pathogens. 

Additionally, vitamin D downregulates the expression of proinflammatory cytokines [1,8,11] and promotes the induction of regulatory T-cells [4]. This leads to attenuation of inflammation and suppression of the cytokine storm, which leads to respiratory distress in COVID-19 patients [8]. A recent Israeli observational study that included more than half a million individuals observed a higher risk of COVID-19 infection in individuals with severe vitamin D deficiency [12]. It is well known that elderly people in residential care homes are at a higher risk of deficiency and are more susceptible to COVID-19 [13]. Therefore, the above observations sparked interest and drew attention once again to the prevalence of hypovitaminosis D.

The seasonal periodicity of autoimmune diseases suggests a possible correlation with 25(OH)D levels. It was observed that reduced maternal sun exposure and the resulting hypovitaminosis D in utero might increase the risk of multiple sclerosis development in offspring born in spring [14]. 25(OH)D levels have also been found to be inversely related to the risk of rheumatoid arthritis [15], possibly due to the suppression of cytokine production and synovial inflammation [16]. Animal studies suggest that vitamin D has immunomodulatory effects on the gut, too [11]. VDR depletion in mice increases inflammation and predisposition to inflammatory bowel disease or enhances its severity [4,11], while vitamin D supplementation could improve the symptoms of patients with inflammatory bowel disease [10]. Deficiency is also linked to increased risk of colorectal cancer [2,11], as well as increased airway hypersensitivity and asthma [17].

Hypovitaminosis D has reached epidemic proportions around the globe, including Southern Europe [18]. Olmos et al., studied a Spanish population sample of 1800 adults over the age of 44 and found a prevalence of insufficiency of about 83% with a significant seasonal variation [19]. In Italy, the rate of 25(OH)D deficiency is estimated to be at 36% [20]. A systematic review of studies, which was conducted in Mediterranean countries and included pregnant women, revealed a prevalence of deficiency of 23–90%, with potential adverse outcomes for maternal and neonatal health [21]. There is an estimated prevalence of 70–100% in the general Indian population [22], while in the USA, breastfed infants who did not receive vitamin D supplementation were found to be severely deficient in winter (78%) [23].

In Greece, 6.6% of a tested sample of Athenian preschool children had vitamin D deficiency with potential adverse developmental outcomes [5]. It has also been found that 57.7% of adults in Athens had insufficient 25(OH)D levels [24]. In Cyprus, this phenomenon has not yet been extensively investigated. In 2015, Kolokotroni et al., published a concerning finding: just 16% of Cypriot adolescents had sufficient 25(OH)D levels, with levels significantly lower in asthmatic children [25]. Surprisingly, in contrast to the above, in Sweden the prevalence of deficiency is possibly less than 1%, as a result of correct policies implemented by the local healthcare services and the fatty fish diet [3].

This pattern of findings has raised concerns regarding the prevalence of vitamin D deficiency in Greece and Cyprus. To our knowledge, there are no available up-to-date data for the vitamin D status of Cypriot adults. Therefore, our findings could potentially lead to increased awareness of the necessity for national health guidelines, targeted and focused on these populations needs.

## 2. Material and Methods

### 2.1. Design of the Study

In this retrospective cross-sectional study, data from 8780 Greek and 2594 Cypriot individuals were blindly collected for an approximately 5-year period (January 2013–October 2017). Data regarding 25(OH)D levels and other patient data including sex, age, and the month at which the blood sample was drawn were collected from the Nuclear Medicine Department of the tertiary healthcare centre AHEPA University Hospital in Thessaloniki (latitude, N 40.7°; longitude, E 22.9°), and a primary healthcare centre in Nicosia, Cyprus “Agios Loukas” (latitude, N 35.1°; longitude, E 33.3°), respectively. Comorbidities were documented in the hospital’s online laboratory information system only for a Greek subgroup of patients and were also blindly collected. Every individual who had a vitamin D measurement at the respective laboratories from January 2013 to October 2017 was included in the study. Individuals with more than three missing data values were excluded.

### 2.2. Vitamin D Laboratory Assays

The Department of Nuclear Medicine at the University Hospital AHEPA of Thessaloniki used a radioimmunoassay (RIA) method by Immunotech & Beckman–Coulter to measure 25(OH)D serum concentrations. Adamou Lab in Nicosia conducted the measurements for the Cypriot database, and quantitative electrochemiluminescence method (ECLIA) was used for the determination of 25(OH)D concentrations using a Cobas 6000 Diagnostics instrument and reagents (Roche Diagnostics, Indianapolis, IN, USA). The laboratory assays used remained unchanged during the study period. Additionally, the technical characteristics (sensitivity, repeatability, intermediate precision, and cross-reactivity) of these two laboratory assays were comparable. Following the Endocrine Society guidelines [26], vitamin D deficiency was defined as 25(OH)D level equal to or below 20 ng/mL and insufficiency 21–29 ng/mL with a preferred level of 30 ng/mL and above.

### 2.3. Statistical Analysis

The statistical analysis was performed in R 3.6.1 (R Core Team (2019), Vienna, Austria). The graphs were produced in R with the package *ggplot2* (Springer-Verlag, New York, NY, USA, 2016). There were two main objectives: first, to assess the levels of 25(OH)D in the two population samples, and second, to examine any demographic factors associated with the observed levels. Univariate (crude) linear regression was used to analyse the relationship between age and 25(OH)D levels. The distribution of 25(OH)D levels was explored via histograms. The results were expressed as mean ± standard deviation (SD) for numerical variables. 

Unpaired *t*-test was used to investigate the relationship between sex and 25(OH)D levels, and ANOVA to investigate the relationship between month or comorbidities and 25(OH)D levels. The assumptions on the normality of residuals for ANOVA and *t*-tests were verified via descriptive measures and plots. Pearson correlation analysis was used to investigate the association of age and 25(OH)D levels. Multivariate two-way ANOVA analysis with independent variables, the month of the year in which the blood sample was drawn, and sex were used to investigate their concurrent association with 25(OH)D levels. An unpaired *t*-test and a linear regression model were used to investigate whether there was a statistically significant difference in 25(OH)D levels between the groups of patients with and without a recorded disease. Linear regression analysis was also performed separately for the disease subgroup of the Greek database. Individuals with any missing values were excluded from the multivariate analysis. The significance level (alpha) was set to 5% and confidence intervals to 95%. A result was considered statistically significant when *p* < 0.05.

## 3. Results

### 3.1. Descriptive Characteristics

#### 3.1.1. Greek Database

**Figure 1:**
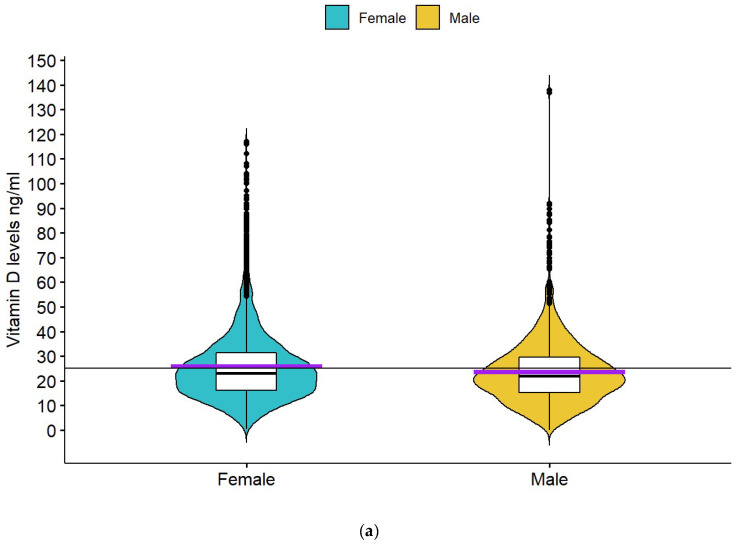

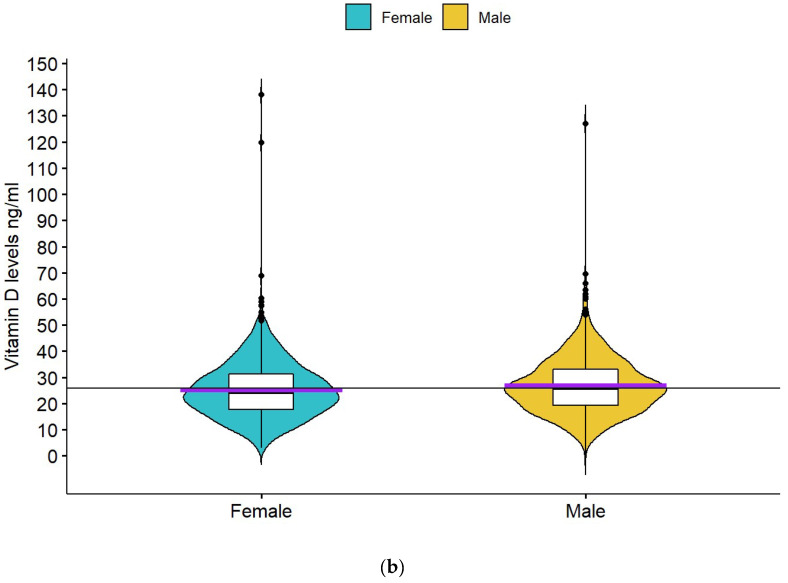
(**a**)Boxplot of 25(OH)D levels according to gender for the Greek database. The short horizontal purple line is the mean, and the short black line represents the median of each group of subjects. The continuous horizontal black line represents the mean for the whole population sample. (**b**) Boxplot of 25(OH)D levels according to gender for the Cypriot Database. The short horizontal purple line is the mean, and the short black horizontal line represents the median of each group of subjects. The continuous horizontal black line represents the mean for the whole population sample.

The Greek database included 8780 subjects over a period stretching from January 2013 to October 2017. Approximately 40% of the sample (N = 3504) had Vitamin D deficiency, and 33% (N = 2913) had insufficiency. The mean levels of 25(OH)D for the total Greek database were 25.08 ± 14.4 ng/mL. The sample included both females (N = 6040, 68.8%) and males (N = 2628, 29.9%). The mean 25(OH)D level for females was 25.7 ± 15.0 ng/mL and for males 23.6 ± 13.0 ng/mL (Figure 1a). For 112 subjects, sex was not recorded.

The database contained subjects from 2 weeks to 96 years old. For 1975 individuals, age was not recorded. The mean age of the subjects for whom age was recorded was 49.3 ± 20.0 years old; 6.6% were children below 12 years old, 2.3% were adolescents between 12 and 17 years old, 81.7% belonged to the 18–74 age group, and 9.4% were elderly of 75 years or above.

Amongst all age groups, elders >75 years old had the lowest mean levels 23.6 ± 13.8 ng/mL, while 12–18-years-old adolescents had the highest mean levels 27.5 ± 14.6 ng/mL. Children below 12 years old had mean levels of 26.3 ± 12.4 ng/mL. 

**Figure 2:**
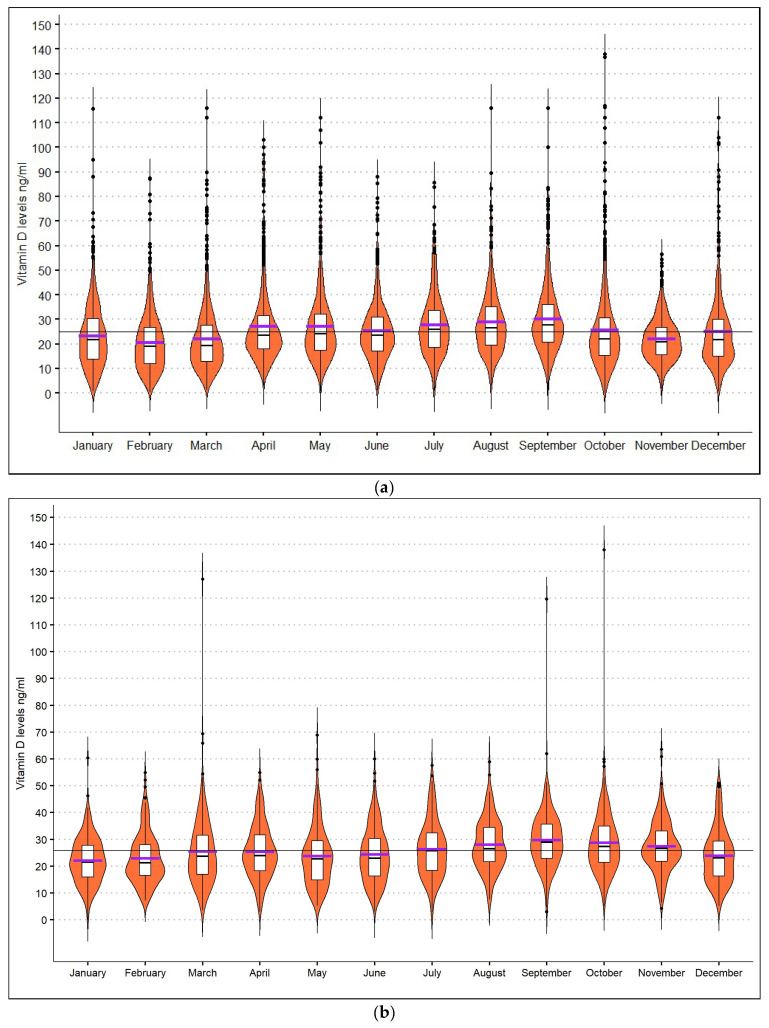
(**a**) Boxplots demonstrating the fluctuance of 25(OH)D levels by month for the Greek database. The purple line represents the mean, and the short black horizontal line the median. The continuous horizontal line represents the mean of the whole Greek population sample throughout the year. (**b**) Boxplots representing the fluctuance of 25(OH)D levels by month for the Cypriot sample. The purple horizontal lines represent the mean, and the black short horizontal lines the median levels for each month. The continuous horizontal line represents the mean of the whole Cypriot population sample throughout the year.

March was the month with the most drawn samples (N = 955), and August had the least (N = 377). September was the month with the highest-recorded mean levels (30.4 ± 17.4 ng/mL), and February was the one with the lowest (20.6 ± 11.9 ng/mL). The fluctuance of 25(OH)D levels by month in the Greek database is presented in Figure 2a.

**Figure 3:**
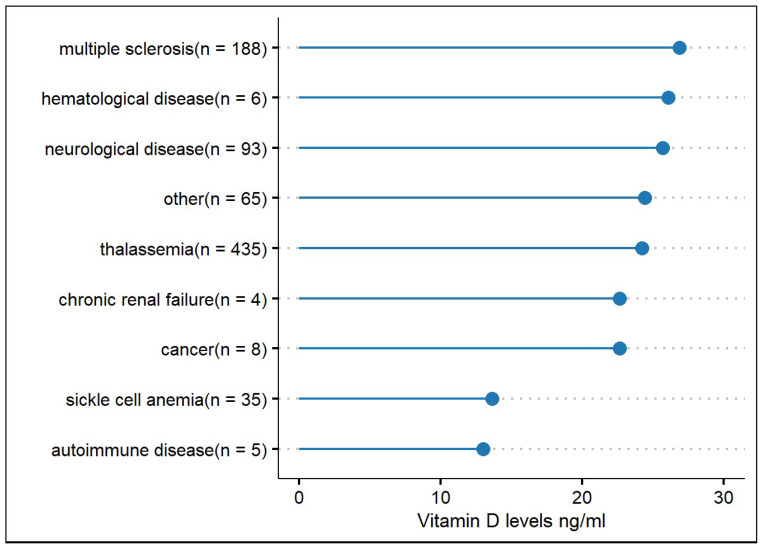
All types of recorded diseases for the subgroup of patients in the Greek database, and the mean Vitamin D levels associated with these. N = the number of subjects for each disease.

In addition to the above, the Greek database had recorded comorbidities for 839 individuals. The mean 25(OH)D level of the total subgroup of patients with a recorded disease was 24.5 ± 14.2 ng/mL. The most recorded diseases were thalassemia 51.8% (N = 435), multiple sclerosis (MS) 22.4% (N = 188), neurological diseases other than MS 11.08% (N = 93) (which included a broad range of neurological diseases such as myopathies, Parkinson’s disease, stroke, neuralgia, mental retardation, dementia, epilepsy, autoimmune diseases, and others), and sickle cell anaemia 4.17% (N = 35). The most frequently recorded in the dataset diseases and the mean 25(OH)D levels are presented in Figure 3. Amongst the subgroup of patients with a recorded disease, those with an autoimmune disorder (for example, Lupus or Chron’s disease) had the lowest 25(OH)D levels (mean 13 ± 8.4 ng/mL), followed by sickle cell anaemia with mean levels 13.6 ± 10.2 ng/mL, and cancer (mean 22.6 ± 9.5 ng/mL). Patients with MS had mean levels of 26.9 ± 15.4 ng/mL and those with other neurological diseases 25.7 ± 15.8 ng/mL. Mean 25(OH)D levels associated with the variables studied for both the Greek and Cypriot sample are presented in Table 1.

#### 3.1.2. Cypriot Database

The Cypriot database included 2594 subjects between September 2013 and May 2017. There was a significant vitamin D insufficiency in the sample studied, with mean 25(OH)D levels 25.8 ± 10.9 ng/mL. Approximately 31% of the individuals had levels less than 20 ng/mL (deficiency), and 38.3% had insufficient levels. Regarding sex, the sample was composed of 1668 (64.3%) females and 907 (34.9%) males. The sex of 19 patients was not recorded. The mean level for females was 25.2 ± 10.8 ng/mL and for males 26.8 ± 10.9 ng/mL (Figure 1b).

The age span of the sample was 2–96 years old. The mean age of the subjects, whose age was recorded, was 39.9 ± 19.4 years old; 9% were children below 12 years old, 11.4% were adolescents between 12 and 17 years old, 75.6% belonged to the 18–74 age group, and 4% were 75 years old or above. For 269 individuals, age was not recorded. September was the month when most samples were collected (N = 301), and August was the one with the least (N = 139). The month with the highest mean 25(OH)D levels was September (mean = 29.7 ± 10.8 ng/mL), and the lowest was January (mean 22.1 ± 8.7 ng/mL). The fluctuance of mean 25(OH)D levels by month for the Cypriot database is presented in Figure 2b.

### 3.2. Associations between Variables and Vitamin D Levels

For the total Greek and Cypriot population sample, the unpaired *t*-test analysis showed that sex was associated with 25(OH)D levels (Greek females 25.7 ± 15.0 ng/mL, males 23.6 ± 13.0 ng/mL, *t* = 6.887, *p* < 0.001; Cypriot females 25.2 ± 10.8 ng/mL, males 26.8 ± 10.9 ng/mL, *t* = −3.67, *p* < 0.001). One-way ANOVA revealed that the month of the year was also associated with 25(OH)D levels in both databases *p* < 0.001. Age was not associated with 25(OH)D levels, as demonstrated by correlation analysis in both samples (Greek: r = −0.03; Cypriot: r = −0.003). The multivariate two-way ANOVA analysis model for the Greek sample showed that month (*p* < 0.001) and sex (*p* < 0.001) were significantly associated with 25(OH)D levels. Similar were the findings of the multivariate two-way ANOVA analysis for the Cypriot sample, where the month (*p* < 0.001) and sex (*p* = 0.002) were significantly associated with vitamin D.

From the above analysis, we obtained contradictory results concerning the association of sex to vitamin D in the studied Greek and Cypriot samples. Females had higher 25(OH)D levels than males in the Greek sample and lower levels than males in the Cypriot sample. Therefore, we performed an unpaired *t*-test analysis for the combined Greek and Cypriot samples to observe the general trend. The mean level of females (Greek and Cypriots combined) was 25.6 ± 14.2 ng/mL, which was statistically significantly higher than that of males, 24.4 ± 12.5 ng/mL (*t* = 4.63, *p* < 0.001), *p* < 0.001 and the result was statistically significant. 

In the linear regression model (R^2^ = 0.09), which only included the subgroup of 839 Greek patients with a recorded disease, the month was not significantly associated with 25(OH)D levels (reference level: September, *p* > 0.05), whereas age (b = −0.14, *p* < 0.001) and sex (reference level Males: b= −5.57, *p* < 0.001) were statistically significantly associated with 25(OH)D levels. In this model, females had lower levels by 5.57 on average compared to the reference value, the males. Age was found to be inversely proportional to 25(OH)D levels for the subgroup of patients with a recorded disease, which was statistically significant. Sickle cell anaemia was the sole disease that was strongly associated with lower vitamin D levels compared to the healthy individuals (b = −11.5, *p* < 0.001) in the linear regression model, which used the ‘healthy’ group as a reference level. Amongst the disease group (reference level: MS), sickle cell anaemia was associated with significantly lower 25(OH)D levels compared to MS (b = −10.8, *p* < 0.001). Lastly, *t*-test analysis showed there was not a statistically significant difference in the mean 25(OH)D levels between the total subgroup of patients with a recorded disease and those without a recorded disease.

## 4. Discussion

The prevalence of vitamin D insufficiency (levels < 30 ng/mL) in the Greek sample was 33% and in the Cypriot sample 38%, while 40% of the Greeks and 31% of the Cypriots had vitamin D deficiency (levels < 20 ng/mL). In the Greek sample, elders had the lowest mean levels at about 24 ng/mL, which is not surprising as cutaneous vitamin D synthesis declines with age [2,27].

Although levels below 30 ng/mL were prevalent in all age groups and age was not found to be significantly associated with 25(OH)D levels, the findings regarding preschool children were most concerning: 31% of preschool children in Greece and 17.6% of preschool children in Cyprus had levels below 20 ng/mL. In our Greek sample, 10.5% had levels < 10 ng/mL, similar to the findings by Nicolaidou et al., in Athenian preschool children, who found a prevalence of 6.6% [5]. In the Cypriot database, adolescents had amongst the lowest levels (25.6 ng/mL), confirming the general trend of previous findings by Kolokotroni et al., who found a mean of 22.9 ng/mL [17].

For both the total Greek and Cypriot samples, the highest mean 25(OH)D levels were recorded in the summer and early autumn, agreeing with other studies conducted in the Mediterranean [18,19] and Greece [24], showing the effect of sun exposure. The highest levels in mother/neonate pairs in Greece were observed during the summer/autumn months [18]. In our study, gender analysis showed opposite associations in the two population samples. In both databases, males comprised about a third of the sample. In the Greek database, females had on average 2.2 ng/mL higher levels than males, but in the Cypriot database, males had on average 1.6 ng/mL higher levels. To investigate this further, the combined database analysis (from both Greece and Cyprus) showed that females had statistically significant higher levels, 25.6 ng/mL, than males, 24.4 ng/mL. However, the Greek sample was larger than the Cypriot; therefore, the combined analysis results could have been influenced by its size. Approximately 70% of the combined sample and 60% of the disease subgroup were females. Our study did not measure vitamin D supplements intake, and women could have been more compliant with this in the Greek database. It is evident that both genders are at risk of hypovitaminosis D, and the difference of 1.6 mg/mL between females and males is most likely to be not clinically significant. Nevertheless, studies suggest that post-menopausal, breastfeeding, and pregnant women, and females covered with a veil have a higher risk of vitamin D deficiency than their male counterparts [21,24,27,28,29].

Although our study did not investigate cultural behaviour, nutrition, or religious beliefs, these have been suggested as potential explanations for the variation of levels in countries with significant levels of sunshine. The modern lifestyle of office work with less sun exposure, despite the darker skin tones, has been suggested as a possible reason [3,18,27]. Vitamin D exerts transcriptional activation and gene repression by binding to VDR [1,2,8]. In an attempt to explain these oxymoronic findings, scientists have investigated the VDR polymorphisms in active, asthmatic Cypriot children. They were found to have lower mean vitamin D levels compared to their non-asthmatic peers [17]. The minor TaqI genotype of VDR was related to asthma in Cypriot adolescents; it has been suggested that those with this genotype and sufficient vitamin D levels are still susceptible to asthma [30]. Similarly, Greek subjects carrying the t TaqI allele presented a two-fold higher risk of developing vitamin D deficiency [31].

Diseases characterised by insufficiency or failure of organs involved in vitamin D metabolism, such as liver and kidney dysfunction, have been associated with vitamin D deficiency [32]. Therefore, it is not a surprise that sickle cell anaemia and thalassemia patients had among the lowest vitamin D mean levels in the studied subgroup. The estimated prevalence of deficiency in patients with sickle cell anaemia globally is 56.4% to 96.4% [33]. 1,25-hydroxyvitamin-D is one of the most potent regulators of cellular growth, and cancer patients reportedly have low levels of vitamin D compared to healthy controls [2].

The low mean levels reported in many Mediterranean and Middle Eastern countries [12,18,19,21,29,34,35] fuelled a debate in redefining the normal range and using a cut off of >20 ng/mL as normal [24]. The Endocrine Society recommends levels >30 ng/mL as optimal. Prospective studies that included patients with MS showed that a 20 ng/mL increment in average serum 25(OH)D levels, even in individuals with sufficient levels, within the first 12 months predicted a 31–57% lower rate of new active lesions [36,37], a 57% lower relapse rate, and lower long-term disability [37]. However, even if we adopt the more relaxed cut-off recommended by the National Institutes of Health in the USA, one-third of both studied samples had inadequate levels of <20 ng/mL. It must be acknowledged that only retrospective data were collected for this study. Therefore, we cannot prove causality, i.e., if vitamin D deficiency preceded the diagnosis of the disease or if vitamin D deficiency resulted from the disease. Future prospective studies are needed to investigate this. 

To our knowledge, this study included the largest Greek population sample investigating the prevalence of hypovitaminosis D to date, and it is the first published study to explore this topic in the adult Cypriot population. Most previous studies in Greece were conducted in adolescents, neonates, and pregnant women [18,21,28,29,38]. A high prevalence of vitamin D deficiency and insufficiency was observed in both samples, confirming the notion of previous observations in Southern European and Mediterranean countries.

The very large sample, and the use of data collected over different seasons for several years, are some of the strengths of this study. However, as it is a retrospective cross-sectional study, it can only suggest an association between variables and vitamin D levels. Other limitations include the lack of simultaneous measurements and collection of parameters related to bone turnover, e.g., parathyroid hormone, calcium, alkaline phosphatase, and lack of data about habits like sun exposure, diet, and skin phenotype. Therefore, further prospective studies are needed to investigate causality between vitamin D levels and associated risk factors.

## 5. Conclusions

Our findings suggest that the abundant sunlight exposure in Greece and Cyprus is not sufficient to prevent hypovitaminosis D. This study showed a significant prevalence of vitamin D deficiency and insufficiency in both population samples. Vitamin D deficiency is more pronounced during the winter and early spring months, and it is encountered in both genders and across every age group. Physicians need to be highly suspicious of hypovitaminosis D in patients with certain autoimmune or haematological diseases who might need surveillance. There is still a need for prospective longitudinal studies to investigate the scale and the biological mechanisms associated with vitamin D and other micronutrients’ deficiencies. Furthermore, interventional studies investigating the beneficial effects of vitamin D supplementation are essential. This might have important implications for public health policies and individualised medicine to avoid a possible resurgence of vitamin D-related diseases, such as rickets, especially with the recent lockdown measures taken by the government to combat the current SARS-COVID-19 pandemic.

## Figures and Tables

**Table 1:** Descriptive characteristics and results for the Greek and Cypriot Database and multivariate analysis model.

	Greek Database N = 8780		Cypriot Database N = 2594	
	(Mean ± SD)	*p*-Value	(Mean ± SD)	*p*-Value
Age (years old)	49.3 (± 20.0)		39.9 (± 19.4)	
Females, N (%):	6040(69)		1668(64)	
Males, N (%):	2628(30)		907(35)	
Vitamin D levels (ng/mL)	25.1 ± 14.4		25.8 ± 10.9	
insufficiency (21–29 ng/mL):	32.8%		38.3%	
deficiency (≤20 ng/mL)	39.9%		31.0%	
Vitamin D levels in each age group (ng/mL)				
<12 years old	26.3 ± 12.4		28.4 ± 8.9	
12–17 years old	27.5 ± 14.6		25.6 ± 8.9	
18–74 years old	25.6 ± 14.9		25.2 ± 10.8	
≥75 years old	23.6 ± 13.8		27.8 ± 17.0	
Vitamin D levels by sex (ng/mL)				
Females	25.7 ± 15.0	*p* < 0.001	25.2 ± 10.8	*p* = 0.002
Males	23.6 ± 13.0		26.8 ± 10.9	
Month with the highest vitamin D mean levels (ng/mL):	September: 30.4 ± 17.4	*p* < 0.001	September: 29.7 ± 10.8	*p* < 0.001
Month with the lowest vitamin D mean levels (ng/mL):	February: 20.6 ± 11.9		January: 22.1 ± 8.7	
Levels of vitamin D for most frequently recorded diseases. Greek database only (ng/mL):				
Thalassemia	24.2 ± 12.9			
Multiple sclerosis	26.9 ± 15.4			
Other neurological diseases *	25.7 ± 15.8			
Sickle cell anaemia	13.6 ± 10.2			

Legend: N = Absolute number of individuals, SD = Standard Deviation, CI = 95% Confidence Interval. * Other neurological diseases group includes a broad range of neurological diseases: demyelinating disorders, myopathies, Parkinson’s disease, stroke, neuralgia, mental retardation, gait disorders, dementia, epilepsy, and autoimmune diseases. *p*-values from the multivariate analysis models are shown in this table for the whole Greek database and the Cypriot database.

## Data Availability

The data that support the findings of this study are available from the corresponding author, [S.X.], upon reasonable request.
